# Targeting tissue factor on tumour cells and angiogenic vascular endothelial cells by factor VII-targeted verteporfin photodynamic therapy for breast cancer *in vitro *and *in vivo *in mice

**DOI:** 10.1186/1471-2407-10-235

**Published:** 2010-05-26

**Authors:** Zhiwei Hu, Benqiang Rao, Shimin Chen, Jinzhong Duanmu

**Affiliations:** 1 Department of Molecular Biophysics and Biochemistry, Yale University School of Medicine, New Haven, CT 06520, USA; 2 Department of Obstetrics, Gynecology and Reproductive Sciences, Yale University School of Medicine, New Haven, CT 06520, USA

## Abstract

**Background:**

The objective of this study was to develop a ligand-targeted photodynamic therapy (tPDT) by conjugating factor VII (fVII) protein with photosensitiser verteporfin in order to overcome the poor selectivity and enhance the effect of non-targeted PDT (ntPDT) for cancer. fVII is a natural ligand for receptor tissue factor (TF) with high affinity and specificity. The reason for targeting receptor TF for the development of tPDT is that TF is a common but specific target on angiogenic tumour vascular endothelial cells (VEC) and many types of tumour cells, including solid tumours and leukaemia.

**Methods:**

Murine factor VII protein (mfVII) containing a mutation (Lys341Ala) was covalently conjugated via a cross linker EDC with Veterporfin (VP) that was extracted from liposomal Visudyne, and then free VP was separated by Sephadex G50 spin columns. fVII-tPDT using mfVII-VP conjugate, compared to ntPDT, was tested *in vitro *for the killing of breast cancer cells and VEGF-stimulated VEC and *in vivo *for inhibiting the tumour growth of breast tumours in a mouse xenograft model.

**Results:**

We showed that: (i) fVII protein could be conjugated with VP without affecting its binding activity; (ii) fVII-tPDT could selectively kill TF-expressing breast cancer cells and VEGF-stimulated angiogenic HUVECs but had no side effects on non-TF expressing unstimulated HUVEC, CHO-K1 and 293 cells; (iii) fVII targeting enhanced the effect of VP PDT by three to four fold; (iii) fVII-tPDT induced significantly stronger levels of apoptosis and necrosis than ntPDT; and (iv) fVII-tPDT had a significantly stronger effect on inhibiting breast tumour growth in mice than ntPDT.

**Conclusions:**

We conclude that the fVII-targeted VP PDT that we report here is a novel and effective therapeutic with improved selectivity for the treatment of breast cancer. Since TF is expressed on many types of cancer cells including leukaemic cells and selectively on angiogenic tumour VECs, fVII-tPDT could have broad therapeutic applications for other solid cancers and leukaemia.

## Background

Accumulating evidence suggests that the receptor tissue factor (TF) is expressed on endothelial cells of pathological blood vessels associated with solid tumours [1-6], wet macular degeneration (wMD) [7,8], and endometriosis[9] but not on endothelial cells of normal blood vessels [1-5,10-13], providing an accessible and specific therapeutic target for these diseases. Because its natural ligand, factor VII (fVII), binds TF with exceptionally high specificity and affinity (up to 10^-12 ^M) [14], we constructed an antibody-like immunoconjugate (Icon) by fusing two fVII peptides to an IgG1 Fc to target TF for the development of a novel immunotherapy [2-4]. Since the binding of fVII to TF would initiate an extrinsic coagulation cascade [15], we introduced a single mutation (K341A) to fVII peptide to reduce its coagulation activity while retaining its binding activity to TF [2,4]. The choice of mutation was based on a previous report [16], in which Dickinson et al. showed that the TF binding activity of K341A mutated fVII was indistinguishable from wild-type fVII, whereas its coagulation function was reduced about eight fold [16]. Delivery of the Icon cDNA by an adenoviral vector, or injection of the Icon protein, resulted in a cytolytic immune attack against the pathological vessels in mouse models of cancer [2-5], wMD [7,8], and endometriosis [9].

Here, we test the use of monomeric fVII peptide to target a laser-activatable photosensitiser to TF for the development of ligand-targeted photodynamic therapy (tPDT) of cells expressing TF, particularly for the angiogenic vascular endothelial cells (VECs) in solid tumours and the choroidal neovasculature of wMD. In the case of cancer, many types of cancer cells (including breast cancer cells) also over-express TF, providing additional target cells for fVII-targeted therapies. A widely used procedure for non-targeted PDT (ntPDT) of wMD involves intravenous injection of the photosensitiser Visudyne followed by irradiation of the eye with a laser emitting 689 nm light, in which Visudyne is activated by the laser light to generate singlet oxygen, resulting in cytoxicity and apoptosis of cells [17]. However, because the non-targeted photosensitiser could be absorbed by normal cells as well as by pathological cells, side effects may be associated with this procedure [18,19]. To improve the safety and efficacy of PDT, tPDT has been proposed and tested by conjugating the photosensitisers to antibodies or ligands for targeting to cells expressing the cognate antigen or receptor [20-23]. In this paper, we target the receptor TF by covalently conjugating its ligand, fVII protein, to VP for the development of a novel and effective fVII-tPDT for the treatment of breast cancer *in vitro *and *in vivo*. Recently, we showed that this fVII-tPDT using the fVII-VP conjugate was also efficacious and safe for the treatment of wMD in a rat model [24].

In the course of the development of TF-targeted therapeutics, Hu and Garen have previously developed fVII(K341A)/IgG1 Fc (Icon) immunoconjugates for immunotherapy of cancer [2-5], wMD [7,8], and endometriosis[9]. Recently, Shoji and co-workers used an active site-inactivated recombinant human fVII (fVIIa) to target EF24, a synthetic curcumin analog, for TF-targeted cancer therapy [25]. To the best of our knowledge, this paper describes, for the first time, the use of fVII to target its cognate receptor TF for the development of ligand-tPDT, which has broad potential for the treatment of cancer and wMD [24].

## Methods

### Cell lines

Chinese Hamster Ovary K1 (CHO-K1, ATCC) cells were grown in F12 medium, murine breast cancer EMT6 and human embryonic kidney 293 cells (kindly provided by Dr. Albert Deisseroth during his tenure at Yale University) in DMEM and human breast cancer MDA-MB-231 cells in RPMI1640 medium supplemented with 10% heat inactivated fetal bovine serum (FBS) (Invitrogen) and 1:100 antibiotics (Sigma). Human umbilical vein endothelial cells (HUVEC) purchased from Yale Vascular Biology and Transplantation Group were grown in M199 (Invitrogen) with 20% heat inactivated FBS, 1:100 antibiotics, and 1:100 Endothelial Cell Growth Supplement solution (ECGS, BD Biosciences).

### Construction of plasmid containing the mfVII cDNAs

The plasmid vector encoding mouse fVII peptide was constructed by PCR amplifying the mouse fVII cDNA with a K341A mutation from a previously constructed Icon (GenBank accession number AF272773) pcDNA3.1(+) plasmid vector as a DNA template [2] and by using high-fidelity Pfx DNA polymerase (Invitrogen) and the following primers: 5'-primer 5'-ACGATCTTAAGCTTCCCCACAGTCTCATCATGGTTCCA-3' and 3'-primer 5'-AGTTTAGCGGCCGCTTAGTGATGGTGATGGTGATGGTGATGGGAGTTCATGTGCTGCCGCTCAAACTTGGCTGCTGCAGTTTCCTTGGATC CCAGTAGTGGGAGT-3'. The amplified mfVII (mfVII-Sp-His) cDNA contains the coding sequence for mfVII (K341A), a BamHI site, ribonuclease S-peptide [Sp (D14N), the first 15 amino acids at the N-terminus of bovine ribonuclease S [26]] and eight histidines (His tag), with a Hind III site at the 5'-end and a Not I site at the 3'-end. The S peptide (Sp or S tag) and polyhistidines (His tag) were designed to be included at the C-terminus of the fVII protein for protein purification and detection. The reason for using the Sp mutant (D14N) instead of wild-type Sp in the fVII protein is that Kim JS and Raines RT showed that Sp (D14N) had a higher affinity for S protein than wild-type Sp [26]. The PCR-amplified cDNAs were sequentially digested with Hind III and Not I and ligated into the pcDNA3.1(+) vector (Invitrogen).

### Production and purification of the mfVII protein

The mfVII plasmid was transfected into CHO-K1 cells using the Superfect transfection reagent (Qiagen), and the transfectant clones were selected with 1 mg/ml G418 (Invitrogen). Individual clones were isolated using Cloning Discs (Sigma) and grown in CHO serum-free medium (SFM) Excel 301 (JRH Biosciences) supplemented with a final concentration of 1 μg/ml Vitamin K1 (Sigma) (for posttranslational modifications, particularly vitamin K-dependent γ-carboxylation of fVII protein) [2-4,27], and the SFM was collected every three to four days. The clone with the highest expression of mfVII protein was selected by Western blotting using anti-His tag antibody (Sigma) and expanded to 10 T175 flasks in F12-complete growth medium with a final concentration of 0.5 mg/ml G418 (Gibco). When the cells reached about 90% confluence, the cells were washed three times with PBS and switched to SFM with Vitamin K1 as described above. The SFM was collected twice a week. After centrifugation to remove the suspended cells, the SFM was pooled and stored at -20°C until affinity purification.

The mfVII was affinity purified using Ni-NTA resin (Qiagen). The purified protein was dialysed against PBS pH7.4 and concentrated to 1 mg/ml using a Millipore Centrifugal device (MWCO 10,000). The affinity-purified mfVII protein was characterised by SDS-PAGE for purity and molecular weight. The mfVII protein was conjugated with photosensitiser as described below or aliquoted and stored at -20°C for future conjugation.

### Extraction and conjugation of Verteporfin to mfVII protein

Chemically pure form of Verteporfin (Benzoporphyrin derivative-monoacid ring A, BPD-MA, molecular weight 718.8) was not available at the time of the study so it was extracted from clinic-leftover liposomal Visudyne (QLT Inc) using an outlined procedure [28] with modifications as follows. Five μl of 6 M HCl was added and mixed by vortexing to 300 μl of Visudyne aqueous solution of clinically reconstituted liposomal formulation, in which the VP concentration was 2 mg/ml. Then, 500 μl of dichloromethane (CH_2_Cl_2_) (Sigma) was added and mixed by vortexing, followed by centrifugation for 5 min. After centrifugation, the organic (VP, lower phase) and aqueous (liposome, upper phase) layers were visibly separated. The VP in the lower phase was carefully transferred to a glass tube and loaded to a freshly prepared silica gel column (Sigma) equilibrated in CH_2_Cl_2_/methanol (3:1). The VP was eluted using CH_2_Cl_2_/methanol (3:1), and the green fractions containing VP were collected, pooled in a glass beaker, aliquoted in 1.5-ml Eppendorf tubes and dried in a SpeedVac. The weight of the VP powder was determined by subtracting the weight of the Eppendorf tube after dissolving and removing VP in dimethylformamide (DMF, Sigma). The VP was adjusted to a final concentration of 10 mg/ml in DMF. A standard curve of VP dye was generated by serially diluting a known concentration of VP in DMF into de-ionised and distilled H_2_O starting from 1000 μg/ml. The equation was *y *= 0.00005 *x *- 0.0008 (*R*^2 ^= 0.9995), where *y *is the OD689 nm at a 1:50 dilution and *x *is the concentration of VP (μg/ml).

VP was conjugated to mfVII protein by EDC (*N*'-3-dimethylaminopropyl-*N*-ethylcarbodiimide hydrochloride, Sigma) as a cross-linker, as previously described [29] but with modifications. VP was activated by mixing 6 μl of DMF, 2 μl of 10 mg/ml VP and 2 μl of a 25-mg/ml solution of EDC in DMF. The 10-μl reaction mixture was incubated at RT for 30 min, followed by the addition of 80 μl of 1 mg/ml mfVII protein in PBS and incubation at RT for 1 hr. For the control, 80 μl of PBS was added to a separate 10 μl-reaction tube. The mfVII-VP conjugate was separated from unconjugated VP on a Sephadex G50 spin column (Roche) following the manufacturer's instructions. After separation, the mfVII-VP conjugate was scanned on a spectrophotometer (Beckman) from 200 nm to 800 nm to measure the protein absorbance at 280 nm and the absorbance at 689 nm for the VP concentration.

### Flow cytometry and cell ELISA for the binding activity of mfVII, mfVII-VP and mfVII/hIgG1 Fc Icon to human and mouse breast cancer cells

TF expression on breast cancer MDA-MB-231 and EMT-6 cells and the non-cancerous CHO-K1 cell line as a normal cell line control was determined by flow cytometry, using mouse Icon (mfVII/hIgG1 Fc) at a concentration of 10 μg/ml followed by a 1:50-diluted anti-human IgG Fc secondary antibody FITC conjugate (Sigma), sorted on a FACS Calibur (BD Biosciences) as described [2-4].

The binding activity of the mfVII and the mfVII-VP conjugate was determined by flow cytometry, similarly as described [2-4], and by cell-ELISA using MDA-MB-231 cells as described below. Briefly, after non-enzymatic dissociation, the cancer cells were blocked in HBSS/10 mM CaCl_2_/1% BSA/0.05% NaN_3 _(FACS buffer as wash and binding buffer), incubated with 20 μg/ml (protein concentration) of mfVII or mfVII-VP conjugate, washed once, incubated with 20 μg/ml goat IgG anti-murine fVII (R & D Systems), washed once, and then incubated with 20 μg/ml secondary anti-goat IgG FITC (Vector Laboratories). After a final wash, the cells were resuspended in HEPES buffer (10 mM HEPES pH7.3, 140 mM NaCl, 10 mM CaCl_2_) supplemented with 2 μg/ml propidum iodide (to distinguish live cells from dead cells) and analysed on a FACS Calibur (BD Biosciences). The control was the same except for omitting the addition of mfVII proteins.

Cell-ELISA was done as follows. MDA-MB-231 cells were grown overnight in 96-well plates at a density of 20,000 cells per well. Then the cells were washed once with pre-warmed TBS-Ca-T (10 mM Tris-HCl pH 7.4, 140 mM NaCl, 10 mM CaCl_2_, 0.1% Tween 20), fixed with 1% paraformaldehyde (J.T. Baker) for 10 min at room temperature, washed three times with TBS-Ca-T buffer, and then blocked with 2% BSA at 37°C for 1 hr. After one wash, the cells were incubated with 0, 0.01, 0.1, 1 and 10 μg/ml mfVII or mfVII-VP conjugate diluted in TBS-Ca-T at 37°C for 1 hr. After three washes, 2 μg/ml goat anti-mfVII (R & D Systems) was added to detect mfVII bound to the cancer cells. After three times washes, 100 μl of OPD substrate solution (Sigma) was incubated at 37°C for 30 min for color development and then was stopped by adding 50 μl 4.5 N H_2_SO4, and A490 nm was read using a microplate reader (Gemini). Binding activity was presented as A490 nm after being subtracted from average A490 nm of 0 μg/ml control wells).

### Confocal imaging of TF expression on HUVECs with or without VEGF stimulation

HUVECs were grown on glass coverslips in growth medium with ECGS in six-well plates. After washing four times with PBS, Human Endothelial SFM (Invitrogen) was added to starve the cells overnight in order to eliminate TF expression due to stimuli in FBS and/or ECGS. A final concentration of 1.1 nM VEGF (BD Biosciences) in the SFM was incubated with the starved HUVECs for four hours to induce TF expression [30,31]. Unstimulated HUVEC controls were also starved but were not incubated with VEGF. Then, both the stimulated and unstimulated HUVECs were fixed with 4% paraformaldehyde for 20 min at RT, washed three times with HBSS-T (HBSS, 0.1% Triton-X 100, 10 mM CaCl_2_), blocked with 1% BSA in HBSS-T at 37°C for one hour, and then incubated with 10 μg/ml monoclonal anti-human TF (American Diagnostica) or 10 μg/ml mouse Icon, followed by an incubation with 1:50 anti-mouse or human IgG FITC (Sigma). To make sure these cells were of endothelium origin, the cells were stained with anti-human CD31 PE conjugate after staining for TF. The coverslips were mounted with anti-fade medium (Molecular Probes) on glass slides and observed and photographed under a confocal microscope (Zeiss L510).

### *In vitro *PDT

Cells were seeded with 10,000 cells per well in 96-well plates and grown overnight in growth medium. The next morning, the cells were incubated with VP, either in unconjugated form or as mfVII-VP conjugate, in HBSS supplemented with 10 mM CaCl_2_, 1% BSA for 90 min at 37°C and 5% CO_2_. The cells were washed once, and 100 μl of complete growth medium was added to each well. The cells were irradiated with a 689 nm laser at various energies (J/cm^2^, 689 nm laser with adjustable power 0-300 mW/cm^2^, B&W Tek Inc). Controls included laser light alone, no treatment and maximal killing control. The cells in the maximal killing control were the same as the no treatment control except that the cells were completely lysed by the addition of a 1/10 volume of 9% Triton X-100 for 45 min prior to the cell viability assay. Duplicate wells were used in all experiments.

### Cell viability by staining with crystal violet and reading absorbance at 595 nm (A595 nm) for loss of monolayer adherence to determine the effect of PDT *in vitro*

The choice of the cell viability assay using crystal violet staining was based on a previous work [32] with modification of reading A595 nm. Mickuvience et al. [32] compared several non-clonogenic assays for determining the effect of PDT on adherent cells in vitro and found that crystal violet staining for measuring the loss of monolayer adherence was the most sensitive assay, as compared to other non-clonogenic assays including [^3^H]-thymidine incorporation, LDH-release, MTT, trypan blue exclusion and CyQUANT.

The viability assay by crystal violet staining was usually carried out at two to three days after PDT, when the cells in control wells reached 95% confluence. Briefly, the cells were washed once with PBS, then fixed with 3:1 mixed methanol: acetic acid at RT for 5 min. The plates were air-dried, and the cells were stained with 0.05% crystal violet in 20% ethanol for 20 min at RT. After staining, the extracellular dye and background were removed by thoroughly rinsing the plates with tap water. The remaining cell-attached dye was dissolved in a 0.1% acetic acid solution in 50% ethanol. The dissolved dye solution was transferred into another 96-well plate, and the A595 recorded on a microplate reader. Blank acetic acid/ethanol solution was added to the 96-well plates as a blank control. Since the A595 nm readings in the maximal killing control wells were always greater than those in the medium alone control well due to crystal violet staining of the remained cell membrane debris, the percent of surviving cells (%) based on the A595 nm was calculated using the formula: *Percent of surviving cells (%) = (experimental-maximal killing average)/(no treatment control average-maximal killing average) *× *100%*.

### Killing mechanisms of VP PDT

After the cancer cells in 96-well plates were treated by tPDT or ntPDT as described above, the plates were centrifuged and half of the culture supernatant in each well was transferred to another 96-well plate for assaying cytotoxicity, while the original plate with the cells containing the other half of the culture supernatant was assayed for cell apoptosis. The Apo-ONE Homogeneous Caspase-3/7 assay (Promega) was used for measuring caspase-3/7 activity and presented relative fluorescent units (relative fluorescent units = experimental fluorescent units - average fluorescent units in no treatment control wells) as evidence of apoptosis, and the CytoTox 96 Non-Radioactive Cytotoxicity Assay (Promega) was used for measuring lactate dehydrogenase (LDH; a stable cytosolic enzyme that is released upon cell lysis and can be used as an indicator of necrosis) in the culture supernatants as evidence of cytotoxicity in the PDT-treated cancer cells following the manufacturer's instructions. The percent of cytotoxicity (%) based on the OD490 nm was calculated using the following formula: *Percent of cytotoxicity (%) = (PDT treated cells-no treatment control average)/(maximal killing control average-no treatment control average) *× *100%*.

### Animal models of mouse and human tumour xenografts and PDT *in vivo*

The animal study protocol was reviewed and approved by the Institutional Animal Care and Use Committee of Yale University. To generate the subcutaneous xenograft model, mouse breast cancer EMT6 cells were subcutaneously injected into female 4-6-week-old Balb/c mice (Taconic Farms Inc) with 1 or 2 × 10^6 ^cells per mouse as described in the figure legends. When the tumour size reached about 100 mm^3^, mfVII-VP or unconjugated VP was intravenously injected into mice at a final concentration of 2 μM VP by an estimate of 1 ml of circulating blood per mouse at a body weight of 20 grams. Control mice were i.v. injected with sterile saline. After 90 min, the tumours were irradiated with 689 nm laser light at 105 J/cm^2 ^while the mice were under anaesthesia with intraperitoneal injection of Ketamine and Xylazine. There were five mice in each group in all animal experiments. The PDT treatment was done a total of four or six times, as indicated in the figure legends. The tumour size was measured in two dimensions with a calliper, and the tumour volume was calculated by the formula: (width)^2 ^(length)/2, as described previously [2-4]. The percentage of tumour growth was calculated by the formula: each individual tumour/its initial volume on day 0 × 100%.

### Statistical analyses

The data on *in vitro *and *in vivo *effects were presented as mean values ± standard deviations using Prism 5 (GraphPad Software) and were analysed for significance between the treated group and the control group using the ANOVA (single factor, one-way with Tukey's Multiple Comparison Test or two-way ANOVA) methods. P values less than 0.05 were considered to be statistically significant. For analyses of statistical significance, duplicate wells in each group were used for all *in vitro *PDT experiments in tissue culture plates and five mice per group were used for the *in vivo *animal studies, unless specified. The equations for half-maximal effective concentrations (EC_50_) were generated and calculated using Microsoft Excel and Prism 5 software.

## Results

### Characterisations of extracted VP, mfVII, and mfVII-VP conjugate

**Figure 1:**
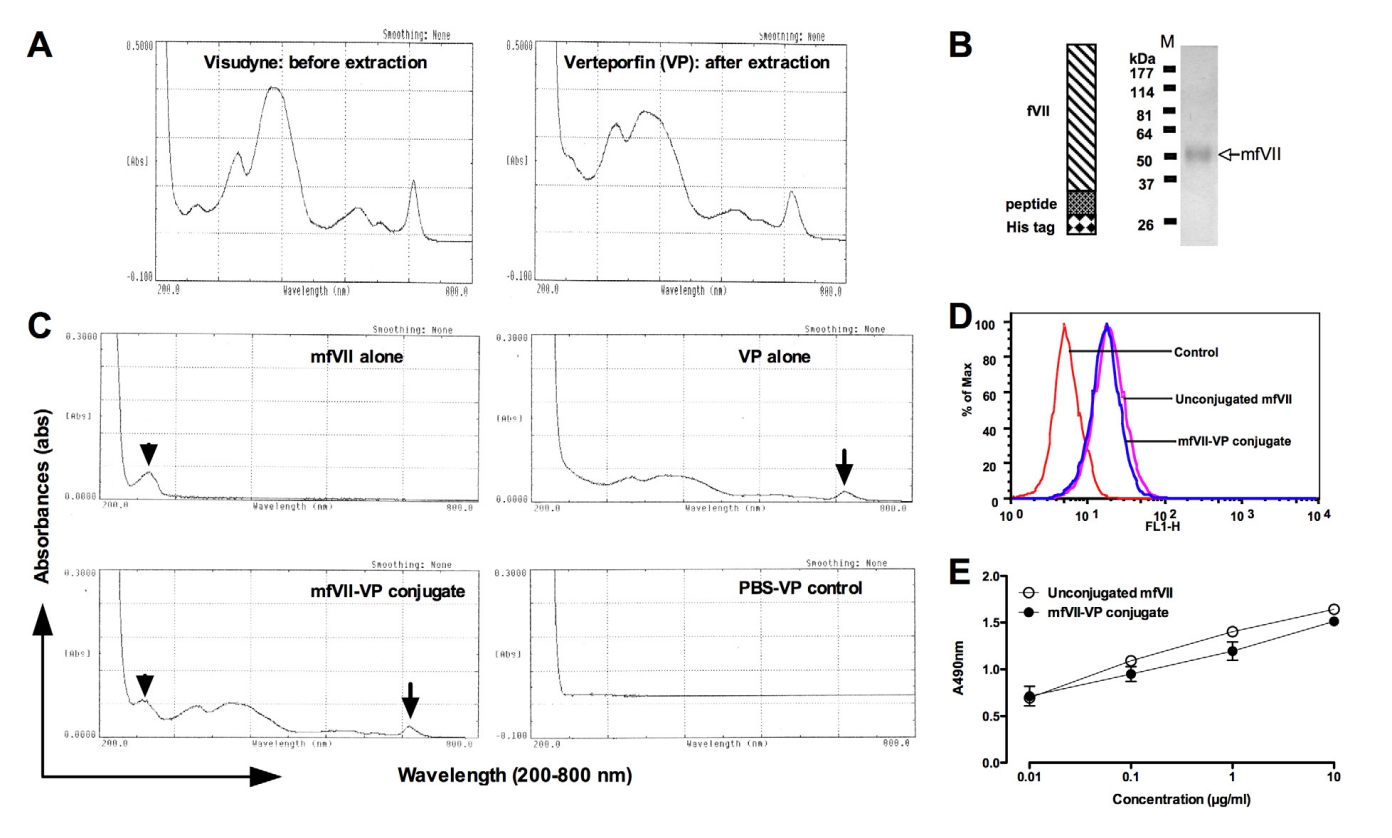
**Veterporfin (VP), mfVII protein, and the conjugate**. **A**. Spectra of VP and Visudyne. **B**. Diagram and molecular weight of mfVII protein determined in SDS-PAGE. fVII, peptide, and His are murine fVII with a mutation of K341A, and S peptide (WT or D14N mutant) and an 8-histidine tag. M represents standard protein markers. **C**. Spectra of VP, mfVII protein, and Sephadex G50 spin column-purified mfVII-VP conjugate and PBS-VP control. Arrows indicate the Q-band of VP and arrowheads indicate the protein absorbance peak. **D and E**. The binding activity of VP-conjugated mfVII and unconjugated mfVII to MDA-MB-231 cancer cells determined by flow cytometric analysis (D) and cell ELISA (E). Results in A-D are representative of two or more experiments.

After extraction, the spectrum of VP is different from that of visudyne before extraction, and the peak at 280 nm disappeared in the spectrum of VP (Figure 1A). More importantly, we observed that free VP dye extracted from visudyne was held in Sephedex G-50 spin columns, whereas visudyne passed the spin columns into collection tubes, indicating that the extraction of VP made the downstream separation of free dye from the conjugated dye possible by Sephedex G-50 spin columns.

The molecular weight of mfVII was about 52.8 kDa, as calculated based on the migration in SDS-PAGE (Figure 1B). After conjugation of mfVII-VP, conjugated VP could be separated from the unconjugated free dye using Sephadex G-50 spin columns. Green solution (VP) was only present in the collection tubes in the reactions containing both VP and protein. In contrast, the green dye was held in the resin of the spin columns in the PBS control reaction, indicating that the VP in the collection tubes was successfully attached to mfVII. Spectrum scanning analyses (Figure 1C) confirmed that the mfVII-VP conjugate had absorbance peaks at both 280 nm (protein peak) and 689 nm (Q-band of VP peaks), whereas free dye and mfVII had a single absorbance peak at 689 nm or 280 nm, respectively. These results indicate that VP was not only successfully conjugated to mfVII but also that free VP was separated from the conjugate. The molar ratio of VP to mfVII protein was 13.1 ± 2.6 (mean ± SD from 15 separate conjugation reaction experiments).

The flow cytometry (Figure 1D) and cell ELISA (Figure 1E) results showed that both the VP-conjugated mfVII and unconjugated mfVII had similar binding activity to breast cancer MDA-MB-231 cells (P = 0.096 by paired t test in Figure 1E), indicating that attaching the dye did not reduce the binding activity of mfVII to the cancer cells.

### TF expression on breast cancer cells and HUVECs

**Figure 2:**
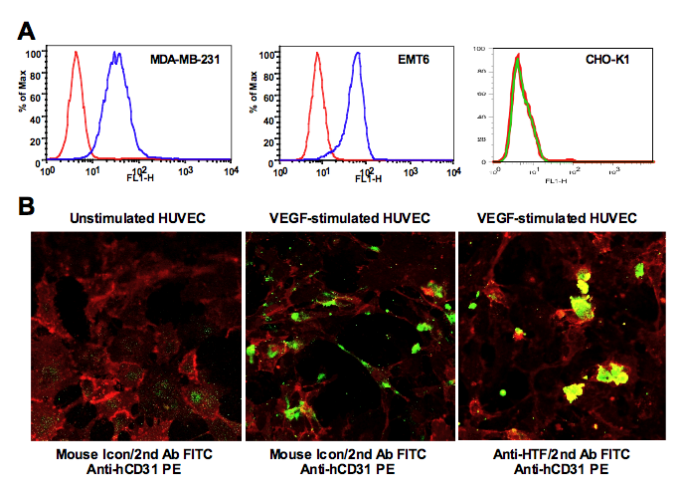
**TF is expressed on tumour cells and VEGF-stimulated HUVECs but not on unstimulated HUVECs**. **A**. TF expression was detected on breast cancer cell lines but not on CHO-K1 cells by flow cytometry using mouse Icon (mfVII/hIgG1 Fc) protein. **B**. Selective expression of TF on VEGF-stimulated HUVECs but not on unstimulated HUVECs was observed and photographed under confocal microscope. HUVECs were stained for TF expression by mouse Icon or anti-HTF antibody (FITC, green) and then by anti-human CD31 PE (red) to verify that TF-expressing cells were of vascular endothelial origin. Results in A and B are representative of two experiments.

As shown in Figure 2, human breast cancer MDA-MB-231 and murine breast cancer EMT6 cells express TF, but CHO-K1 cells do not (Figure 2A). The reason for the choice of a Hamster cell line (CHO-K1) as a TF-negative cell control is because there were very few immortalised normal human cells for potential use as the TF-negative control, one of which used in this study was human embryonic kidney 293 cell line provided by Dr. Albert Deisseroth. In addition, TF expression was only detected by both mfVII/hIgG1 Fc Icon and anti-TF antibody on VEGF-stimulated HUVECs but not on unstimulated HUVECs (Figure 2B), indicating that TF is specifically expressed on angiogenic VECs, such as those in the tumour neovasculature of human breast cancer from patients [1] and human melanoma tumor xenografts from mice [2].

### fVII-targeting improved the selectivity for VP PDT

**Figure 3:**
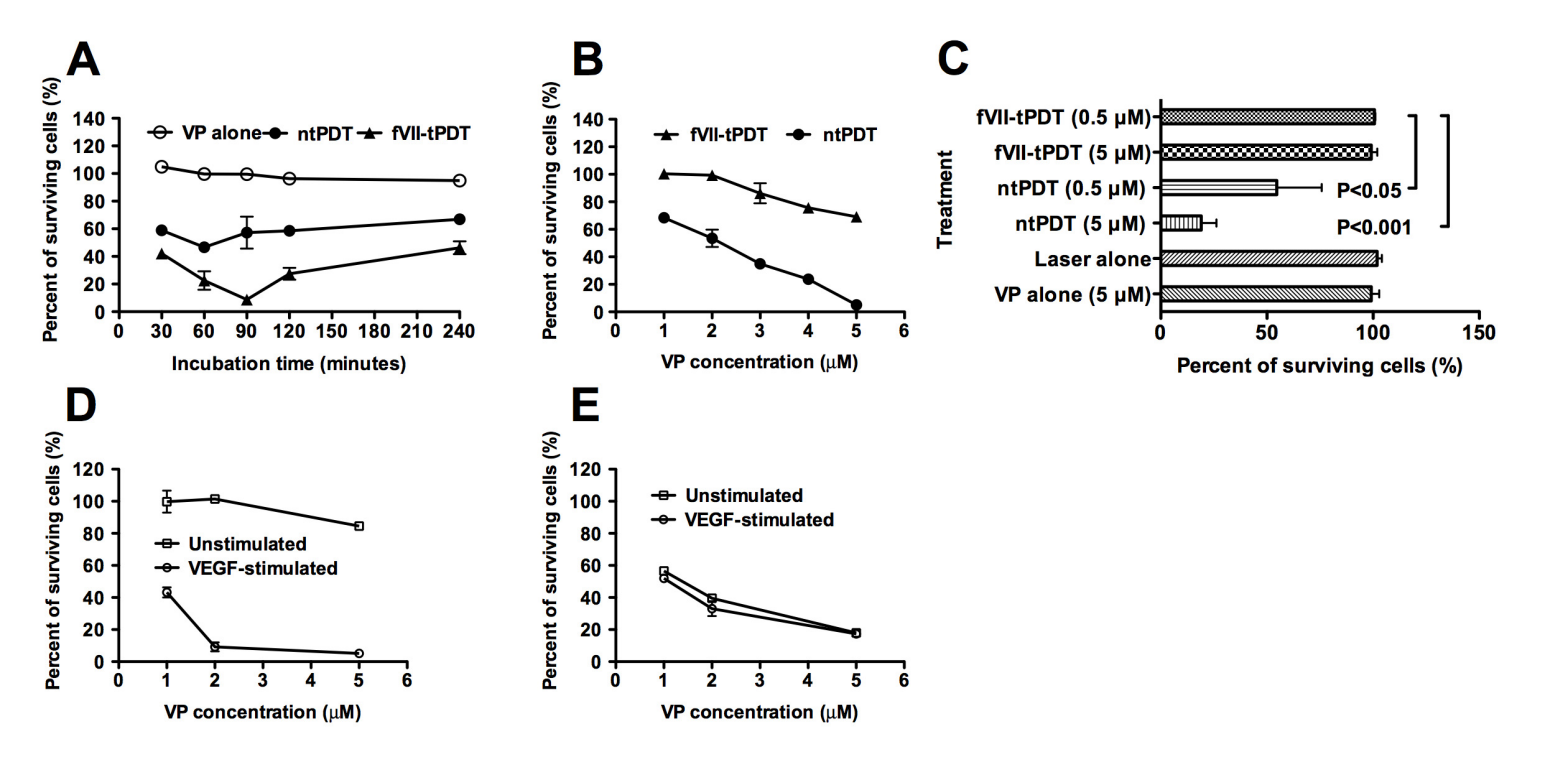
**fVII targeting improves the selectivity of VP PDT**. **A**. Incubation time of fVII-VP and free VP affected the effect of fVII-tPDT and ntPDT (2 μM, 689 nm laser at 60 J/cm^2^) on killing MDA-MB-231 cancer cells. However, VP alone without laser irradiation (VP alone) did not have an effect on killing the cancer cells. **B**. Comparison of fVII-tPDT and ntPDT (689 nm laser at 60 J/cm^2^) for side effects on non-TF-expressing CHO-K1 cells as a normal cell line. **C**. VP PDT (fVII-tPDT or ntPDT with 0.5 or 5 μM VP at 60 J/cm^2^) for the non-TF expressing 293 line as a normal cell line control. **D and E**. fVII-tPDT and ntPDT (689 nm laser at 36 J/cm^2^) for HUVEC with or without induction of TF expression by VEGF, as seen in Figure 2B. Results in A-D are representative of two experiments.

We first optimised the incubation time and showed that the strongest killing effect of fVII-tPDT (2 μM VP and 60 J/cm^2^) on human breast cancer MDA-MB-231 cells occurred when the incubation time with fVII-VP conjugate was 90 min (Figure 3A). Therefore, we decided to use 90 min as the drug (fVII-VP conjugate)-laser light interval.

Then, we showed in Figure 3B that 2 μM VP with fVII-tPDT had no effect on non-TF expressing CHO-K1 cells (Figure 2A), as an example of a normal cell line, whereas ntPDT had the side effect of killing CHO-K1 cells even at 1 μM VP concentration (P < 0.001 for fVII-tPDT vs. ntPDT at each VP concentration by two-way ANOVA). Furthermore, fVII-tPDT (60 J/cm^2^) even at 5 μM did not have any side effects on a non-TF expressing 293 cell line (Figure 3C) (P > 0.05 vs. controls by one-way ANOVA with Tukey's Multiple Comparison Test), whereas ntPDT (60 J/cm^2^) had significant effect on 293 cells even at 0.5 μM (Figure 3C) (P < 0.05 or < 0.001 for 0.5 μM and 5 μM ntPDT as compared to controls and 0.5 μM fVII-tPDT, respectively, by one-way ANOVA with Tukey's Multiple Comparison Test). Note that VP and laser alone did not have any toxicity to the cancer cells (Figure 3A) and normal 293 cells (Figure 3C). We conclude that fVII targeting significantly improves the selectivity of VP PDT.

To determine the effect and selectivity of tPDT for tumour angiogenic VEC, we used HUVEC as normal VEC control, which does not express TF until being stimulated with VEGF (Figure 2B), a potent angiogenic growth factor. We showed that fVII-tPDT at 2 μM VP and a light dose of 36 J/cm^2 ^killed almost 90% of VEGF-stimulated HUVEC cells (Figure 3D), which represent angiogenic tumour VECs in pathological angiogenesis, but had no effect on unstimulated HUVEC cells (Figure 3D) (P < 0.001 at all three VP concentrations for VEGF-stimulated vs. unstimulated by two-way ANOVA), which do not express TF and represent normal VECs (Figure 2B). In contrast, ntPDT even at 1 μM VP and the same light dose (36 J/cm^2^) killed both the VEGF-stimulated and the unstimulated HUVEC cells without any selectivity (Figure 3E) (P > 0.05 for VEGF-stimulated vs. unstimulated by two-way ANOVA), suggesting that fVII targeting indeed improved the PDT selectivity between normal and pathological vascular endothelial cells. Since 2 μM VP in fVII-tPDT did not have any effect on killing normal cell lines in both cases of CHO-K1 and unstimulated HUVEC cells, the VP concentration of 2 μM was chosen in the efficacy tests of fVII-tPDT *in vitro *and *in vivo *thereafter. Taken together, these results indicate that tPDT by fVII to TF could not only distinguish tumour cells from normal cells but could also distinguish tumour angiogenic VECs from normal quiescent VECs.

### Improved effect of fVII-targeted PDT as compared to ntPDT

**Figure 4:**
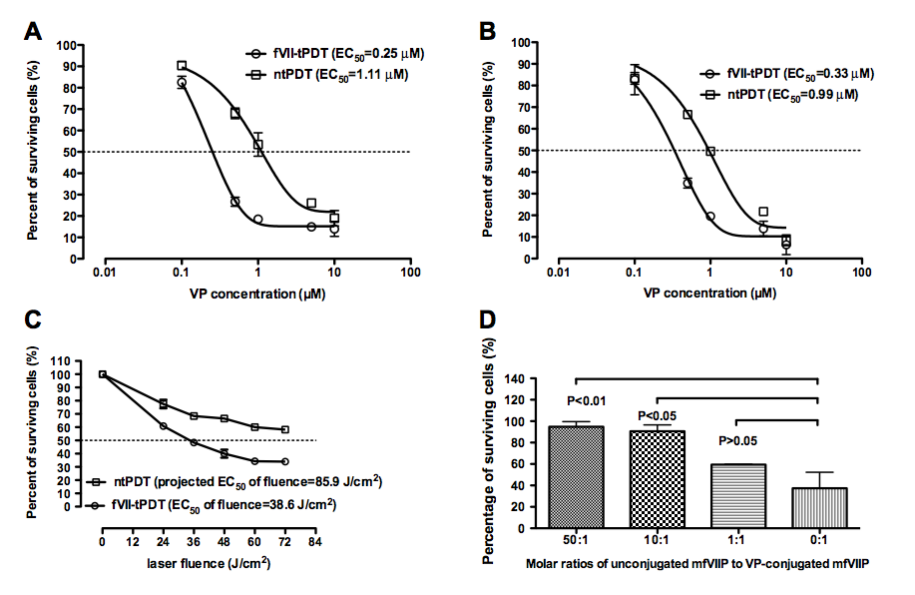
**fVII targeting enhances the therapeutic effect of VP PDT for human and mouse breast cancer cells**. **A and B**. fVII targeting decreased the EC_50 _(crossing with dotted lines) concentration of VP in fVII-tPDT by three to four fold for human (MDA-MB-231) (A) and mouse (EMT6) (B) breast cancer lines, compared to ntPDT (689 nm laser for 60 J/cm^2^). **C**. fVII targeting also decreased the EC_50 _(crossing with dotted line) of the laser energy for VP PDT (2 μM VP). **D**. The effect of fVII-targeted VP PDT (2 μM VP, 689 nm laser for 60 J/cm^2^) could be inhibited by unconjugated mfVII. Results in A-C are representative of two experiments.

The PDT results with breast cancer cell lines showed that the effect of tPDT was stronger than those of ntPDT on killing both human and mouse breast cancer MDA-MB-231 and EMT6 cells with a VP concentration-dependent response and that fVII targeting decreased by about three to four fold the EC_50 _concentration of VP (Figure 4A-B), indicating that fVII targeting improved the effect. Moreover, when the VP concentration was 2 μM, the EC_50 _of the laser energy in tPDT was about half of that in ntPDT (Figure 4C), further indicating that fVII targeting could also reduce laser exposure, which could decrease the risk of side effects of ntPDT.

As shown in Figure 4D, the effect of fVII-tPDT, presented as percent of surviving cells, was partially inhibited at a ratio of 1:1 (unconjugated mfVII: VP-conjugated mfVII) (59.4 ± 0.4%) and almost completely inhibited at ratios of 10:1 and 50:1 (90.6 ± 5.9% and 94.8 ± 4.7%, respectively), as compared to no addition of mfVII (cell survival percent was 37.4 ± 14.8% at the ratio of 0:1) (P > 0.05, < 0.05 and < 0.01 for 0:1 vs. 1:1, 10:1 and 50:1, respectively, by one-way ANOVA), indicating that the effect was mediated by mfVII binding to TF.

### Killing mechanisms induced by VP PDT

**Figure 5:**
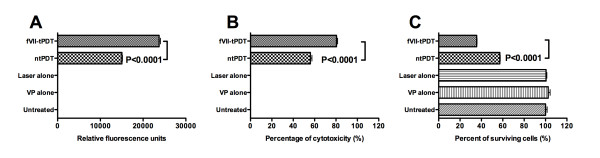
**fVII-tPDT induces stronger levels of apoptosis and cytotoxicity (necrosis) than ntPDT in human breast cancer MDA-MB-231 cells**. Both fVII-tPDT and ntPDT were carried out at 2 μM VP and 60 J/cm^2^. **A**. Apoptosis assay for Caspase 3/7 activity in the PDT-treated cancer cells. **B**. Cytotoxicity assay for LDH release from PDT-treated cancer cells into the culture medium. **C**. PDT therapeutic effect assayed by crystal violet staining.

The results in Figure 5 show that both apoptosis and cytotoxicity were induced in the MDA-MB-231 cancer cells treated by tPDT and ntPDT, but tPDT induced significantly stronger apoptosis (Figure 5A) and cytotoxicity (Figure 5B) than ntPDT (P < 0.0001 by one-way ANOVA with Tukey's Multiple Comparison Test), which was consistent with the results of therapeutic effect as determined by clonogenic assay (Figure 5C) (P < 0.0001). The Caspase 3/7 activity was the highest at 12 hrs compared to those at 8 and 16 hrs after PDT, and the LDH activity in the cytotoxicity assays was higher at 1 hr than that at 4 hrs after PDT.

### Effect of tPDT on cancer *in vivo *in mice bearing mouse breast cancer

**Figure 6:**
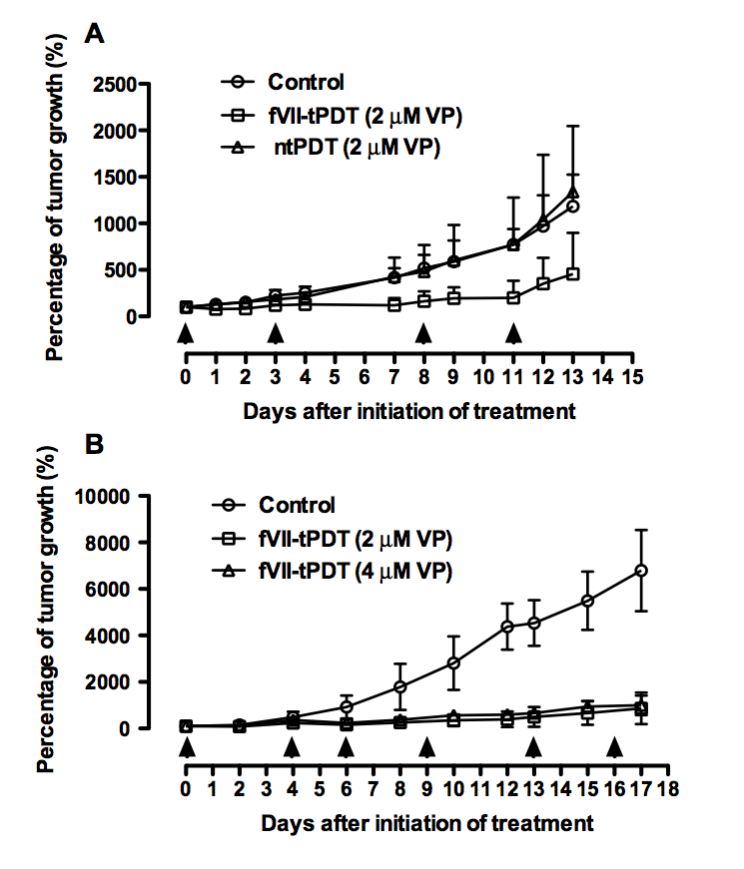
**fVII targeting improved the effect of VP PDT for the treatment of murine breast cancer EMT6 in female Balb/C mice**. Mouse breast cancer EMT6 cells were subcutaneously injected into female 4-6-week-old Balb/c mice (Taconic Farms Inc) with 1 × 10^6 ^(A) or 2 × 10^6 ^(B) cells per mouse. When the tumour size reached about 100 mm^3^, the mice were treated as follows. **A**. fVII-VP tPDT and ntPDT with 2 μM VP and a 689 nm laser for 105 J/cm^2 ^were carried out on days 0, 3, 8, and 11 (arrows). **B**. fVII-tPDT with a final concentration of 2 or 4 μM VP in mfVII-VP was carried out with a 689 nm laser at 105 J/cm^2 ^on days 0, 4, 6, 9, 13, and 16 (arrows). Control mice were i.v. injected with PBS buffer.

As shown in Figure 6A, fVII-tPDT at 2 μM of VP was effective in inhibiting the tumour growth of murine breast cancer in mice (P < 0.01 vs. control by one-way ANOVA with Tukey's Multiple Comparison Test) (3-4 mice per group in Figure 6A). In contrast, ntPDT did not have any effect (P = 0.979 by ANOVA single factor). Moreover, the results in Figure 6B show that tPDT with either 2 μM or 4 μM VP had significant therapeutic effects as compared to controls (P < 0.001 vs. control by one-way ANOVA with Tukey's Multiple Comparison Test). However, increasing the VP concentration from 2 μM to 4 μM did not significantly further enhance the efficacy (P = 0.744 by ANOVA single factor) (five mice per group in Figure 6B), indicating that tPDT with 2 μM VP in the mfVII-VP conjugate reached the optimal therapeutic potential under the experimental conditions (Figure 6B), probably due to the saturation of the binding of the target cells by fVII in the fVII-VP conjugate. The mice were observed for any signs of toxicities during the experiments and examined morphologically at the time of euthanisation. All of the mice, including the controls and the PDT-treated mice, in the experiment in Figure 6A were normal, and no metastases were found in any of the mice. In the experiment in Figure 6B, all of the control mice had enlarged spleens and one mouse had liver and peritoneal metastases. All of the mice treated by fVII-tPDT had no enlarged spleens, but one mouse in the 4 μM fVII-tPDT group had peritoneal metastases, although its subcutaneous tumour responded to the fVII-tPDT treatment, suggesting that fVII-tPDT as a localised therapy had a limited ability to penetrate and therefore had no effect on metastatic or internal tumours located beyond the penetration ability of the 689 nm laser light. No other signs of side effects were observed in these mice.

In addition, whole mouse body weights were measured during the period of the experiments, and no differences and no losses of body weight between the control groups and the treatment groups were observed, indicating that the tPDT procedure under the studied conditions was not toxic to mice.

## Discussion

Photodynamic therapy is a treatment involving three key components: a photosensitiser, light, and tissue oxygen. It can be used as a stand-alone modality or in combination with surgery, radiotherapy, chemotherapy, or anti-angiogenic therapy [33] for cancer. However, ntPDT can cause severe side effects due to the poor selectivity of photosensitisers [18,19]. To overcome the poor selectivity and improve the effect, targeting of the photosensitisers with antibody and ligand has been tested [20-24,34-36], with the hope of specifically targeting the diseased cells and then internalising the photosensitisers into the target cells.

In this paper, we target the receptor TF for the development of a novel fVII-targeted verteporfin PDT. The reason for targeting TF is based on its over-expression in many types of cancer cells, including solid cancers [1-6,12,25,37] and leukaemia [38-40], and very importantly its selective expression in pathological neovascular endothelial cells in cancer [1,2,4], choroidal neovasculature [7,8] and endometriosis [9] but not on normal VECs [1,2,4,7,8]. Garen and Hu have targeted TF using its natural ligand fVII for the development of Icon (fVII/IgG1 Fc) immunotherapy [2-4], which showed dramatic efficacy for treatment of several types of cancer [2-5], wMD [7,8] and endometriosis [9] in animal models. The results in Figure 2B confirmed that TF expression was only detected on VEGF-stimulated angiogenic HUVECs but not on unstimulated quiescent normal HUVECs. The results in Figure 2B also showed that fVII-containing mouse Icon could bind to angiogenic HUVECs but not normal resting HUVECs, providing the basis and rationale that fVII-tPDT that could selectively kill angiogenic VECs but have no side effects on unstimulated HUVECs (Figure 3D), whereas ntPDT could not distinguish angiogenic VECs from normal VECs for killing (Figure 3E). The reasons for using fVII instead of making antibodies to TF are that: (i) the affinity (Kda) of fVII to TF (10^-12 ^M) [14] is far higher than that of antibodies to TF (10^-8 ^to 10^-9 ^M)[41,42], and (ii) therapeutic and diagnostic fVII-containing proteins can be made in human sequence by DNA recombinant technologies for future clinical trials and usages in murine sequence for tests in preclinical studies.

To develop fVII-tPDT, we first tested and conjugated dimeric Icon protein (fVII/IgG1 Fc, molecular weight ~210 kDa, unpublished data) with various photosensitisers in our preliminary studies. For better penetration into tumour tissues, we decided to make a smaller monomeric fVII protein fused with S-tag [26] and His-tag (molecular weight = 52,800 daltons, Figure 1B). Both the S-tag and His-tag can be used for purification and detection. In addition, since the conjugation of photosensitisers to targeting antibodies or ligands could possibly reduce or even abolish the binding activity of targeting proteins, Alan Garen and Zhiwei Hu proposed to use S protein/S peptide [26] as a delivery system for the development of targeted PDT, in which S peptide is synthesised with the targeting protein molecules, e.g., mfVII in this case, by a recombinant DNA technique and then commercially available S protein is conjugated with the photosensitisers (unpublished data). Since conjugation of VP to mfVII did not affect the fVII binding activities to cancer cells (Figure 1D-1E), it was not necessary to use the S protein/S peptide system as the two components delivery system for the development of VP tPDT in this paper. Nevertheless, this S protein/S peptide system could be useful for those targeting molecules if their binding activities are reduced or abolished after direct conjugation with photosensitisers for tPDT.

Visudyne is approved for the treatment of wet macular degeneration in clinical settings and is being tested as ntPDT for cancer in preclinical studies [43]. In our pilot studies, we first conjugated fVII protein with Visudyne using EDC, which had been used in combination with NHS (*N*-hydroxysuccinimide) to conjugate a scFv antibody to SnCe6 [29]. We observed that free Visudyne could not be separated from the conjugated dye by Sephadex G50 spin columns. This indicated that the other components in the liposomal formulation of VP could interfere with the separation of unconjugated VP from the conjugated dye. Therefore, it was necessary to extract the active component of benzoporphyrin derivative (BPD-MA) from Visudyne, which is referred to here as VP. When using the extracted VP, free VP was held in the size exclusion Sephadex G50 spin columns when PBS buffer alone was added to the EDC-activated VP reaction (PBS-VP control in Figure 1C). This was the reason for the lack of a Q-band absorbance peak in the PBS-VP control after being separated by the spin column (Figure 1C). A better source of VP for the conjugation reaction, of course, is a chemically synthesised pure formulation of VP (BPD-MA), which was not available to us at the time when we carried out the experiments reported here.

Nevertheless, VP either extracted from Visudyne or synthesised in pure formulation has been targeted with homing peptide for VEGF receptor or single chain Fv antibodies for treatment of wMD [28] and cancer [34,36], respectively. Although those previous papers used a different conjugation procedure, the molar ratio of VP to scFv was 14.1:1 [34,36], which is similar to the ratios of VP to mfVII (13.1 ± 2.6:1, mean ± SD from 15 separate conjugation reactions). However, Bhatti et al. noticed that there was 30% of non-covalent binding VP present in the final conjugates [36], possibly due to inefficient separation of free dye by the use of a dialysis procedure. Considering that the molecular weight of mfVII protein (52.8 kDa) is bigger than that of scFv (30 kDa) [36], our results of VP-to-fVII ratios were not surprising. Bhatti et al. also found that the IC50 of VP (1.4 μM) in anti-HER2 C6.5 scFv-targeted VP PDT was about four-fold less than that (5.4 μM) in free VP PDT for the HER 2+ SKOV-3 ovarian cancer line, indicating that scFv targeting improved the effect of VP PDT for that cancer line *in vitro *by about four fold. Their observations were similar to our results, although the EC_50 _of VP in fVII-tPDT (0.25 μM for human breast cancer MDA-MB-231 and 0.33 μM for murine breast cancer EMT6 in Figure 4A-4B) was about one fifth or one third of that (1.4 μM) in the previous paper [36]. *In vivo *studies were not done with scFv-VP or free VP PDT in the previous papers [34,36]. In this paper we showed that fVII-tPDT had a significantly stronger effect than ntPDT for the treatment of breast cancer in mice (Figure 6A). The reason that ntPDT did not have the effect *in vivo *but had the effect *in vitro *is probably because liposomal components had been removed, so that the transportation of liposome-free VP to the tumour mass by intravenous injection was not as efficient as that of the liposomal formulation of Visudyne, although there was no problem for transportation because VP was directly incubated with the test cells. Further increasing the VP concentration in the fVII-VP conjugate did not further increase the effect of fVII-tPDT in the current setting, probably because fVII even at the lower concentration had reached the saturation binding to the tumour vasculature. Therefore, it is possible to get stronger effect by increasing the laser light fluence (but not VP concentration) in future experiments. Taking the *in vitro *and *in vivo *results reported in this paper together with the major findings in our recent paper on fVII-tPDT for wMD [24], we conclude that this novel fVII-targeted VP PDT improves the selectivity and efficacy of free VP PDT for the treatment of breast cancer and wMD and induces apoptosis and necrosis as the underlining mechanisms of action.

For future clinical trials, we have constructed human fVII proteins with a lys341Ala mutation, similarly by recombinant DNA technology, for fVII-tPDT for patients with cancer or wMD. To establish additional proof of principle, our laboratory has developed and tested another fVII-tPDT using another potent photosensitiser, Sn(IV) chlorin e6, and the results showed that fVII-targeted SnCe6 PDT was more selective and also safe for the treatment of breast and lung cancer *in vitro *and *in vivo *in mice (Hu et al. Breast Cancer Research and Treatment. In press).

## Conclusions

In this paper we describe for the first time the development of a novel and effective ligand-targeted PDT for breast cancer by conjugating fVII, a natural ligand with high affinity and specificity for TF, with the US FDA-approved photosensitiser veterporfin. Since TF is specifically expressed on angiogenic tumour VECs [1-5] and is also overexpressed by many types of cancer cells including solid cancers [1-6,12,25,37] and leukaemia [38-40], and since fVII-targeted VP PDT showed a stronger effect and better selectivity than free VP PDT for breast cancer *in vitro *and *in vivo*, we anticipate that this fVII-VP tPDT could have broad therapeutic potential for primary and metastatic tumours, not only for breast cancers but also for other tumours, as long as these tumours are laser light-accessible and express TF on their tumour cells and/or tumour VECs.

## Abbreviations

fVII: coagulation factor VII; TF: Tissue factor; VP: Verteporfin; mfVII: Mouse factor VII protein; Sp: S peptide or S tag; His tag: Polyhistidine tag; PDT: Photodynamic therapy; tPDT: Targeted photodynamic therapy; ntPDT: Non-targeted photodynamic therapy; wMD: The wet form of macular degeneration; CNV: Choroidal neovascularisation; HUVEC: Human umbilical vein endothelial cell; VEC: Vascular endothelial cell.

## Competing interests

The authors declare that they have no competing interests.

## Authors' contributions

ZH designed the experiments and wrote the paper. ZH, BR, SC and JD performed the experiments and analysed the data. All authors read and approved the final manuscript.

## Authors' information

Current address for Benqiang Rao: Sun Yat-Sen University, The Sixth Affiliated Hospital, Guangzhou, China; and for Jinzhong Duanmu: Nanchang University, the First affiliated Hospital, Nanchang, China.

## Pre-publication history

The pre-publication history for this paper can be accessed here:

http://www.biomedcentral.com/1471-2407/10/235/prepub
